# Complete mucosal healing of distal lesions induced by twice-daily budesonide 2-mg foam promoted clinical remission of mild-to-moderate ulcerative colitis with distal active inflammation: double-blind, randomized study

**DOI:** 10.1007/s00535-017-1376-4

**Published:** 2017-08-04

**Authors:** Makoto Naganuma, Nobuo Aoyama, Tomohiro Tada, Kiyonori Kobayashi, Fumihito Hirai, Kenji Watanabe, Mamoru Watanabe, Toshifumi Hibi

**Affiliations:** 1 Department of Gastroenterology and Hepatology, School of Medicine, Keio University, Shinanomachi 35, Shinjuku-ku, Tokyo, 160-0016 Japan; 2 Gastrointestinal Endoscopy and Inflammatory Bowel Disease Center, Aoyama Medical Clinic, Hyogo, Japan; 3 Tada Tomohiro Institute of Gastroenterology and Proctology, Saitama, Japan; 4 Department of Research and Development Center for New Medical Frontiers, School of Medicine, Kitasato University, Kanagawa, Japan; 5 Department of Gastroenterology, Fukuoka University Chikushi Hospital, Fukuoka, Japan; 6 Department of Gastroenterology, Osaka City General Hospital, Osaka, Japan; 7 Department of Gastroenterology and Hepatology, School of Medicine, Tokyo Medical and Dental University, Tokyo, Japan; 8 Center for Advanced IBD Research and Treatment, Kitasato Institute Hospital, Kitasato University, Tokyo, Japan

**Keywords:** Ulcerative colitis, Budesonide foam, Mucosal healing

## Abstract

**Background:**

Budesonide foam is used for the topical treatment of distal ulcerative colitis. This phase III study was performed to confirm mucosal healing and other therapeutic effects of twice-daily budesonide 2-mg foam in patients with mild-to-moderate ulcerative colitis including left-sided colitis and pancolitis.

**Methods:**

This was a multicenter, randomized, placebo-controlled, double-blind trial. A total of 126 patients with mild-to-moderate ulcerative colitis with active inflammation in the distal colon were randomized to two groups receiving twice-daily budesonide 2 mg/25 ml foam or placebo foam. The primary endpoint was the percentage of complete mucosal healing of distal lesions (endoscopic subscore of 0) at week 6. Some patients continued the treatment through week 12. Drug efficacy and safety were evaluated.

**Results:**

The percentages of both complete mucosal healing of distal lesions and clinical remission were significantly improved in the budesonide as compared with the placebo group (*p* = 0.0003 and *p* = 0.0035). Subgroup analysis showed similar efficacy of budesonide foam for complete mucosal healing of distal lesions and clinical remission regardless of disease type. The clinical remission percentage tended to be higher in patients achieving complete mucosal healing of distal lesions than in other patients. There were no safety concerns with budesonide foam.

**Conclusions:**

This study confirmed for the first time complete mucosal healing with twice-daily budesonide 2-mg foam in mild-to-moderate ulcerative colitis with distal active inflammation. The results also indicated that complete mucosal healing of distal lesions by budesonide foam promotes clinical remission of ulcerative colitis. *Clinical trial registration no.*: Japic CTI-142704.

**Electronic supplementary material:**

The online version of this article (doi:10.1007/s00535-017-1376-4) contains supplementary material, which is available to authorized users.

## Introduction

Ulcerative colitis (UC) is a chronic condition with complaints including increased stool frequencies, rectal bleeding, and abdominal pain. UC is associated with continuous lesions from the rectum and is classified into proctitis, left-sided colitis, and pancolitis subtypes. The inflammation of UC is characterized by initial onset in the rectum and subsequent adoral progression with time. Even among patients with pancolitis [1–3], severe and intractable distal inflammation is not uncommon. It is therefore very important to ameliorate the symptoms of distal inflammation not only in patients with proctitis but also in those with pancolitis and left-sided colitis.

The Mayo endoscopic subscore has frequently been used for the evaluation of mucosal healing, though no validated definition has been established [4]. The Mayo scoring system evaluates the mucosal condition according to a 4-point scale from 0 to 3 based on, e.g., the presence of erythema, friability, ulceration, and erosion. The score of 0 corresponds to normal mucosa. Mucosal healing was defined as a Mayo endoscopic subscore of 0 or 1 in many previous clinical studies [5]. Completion of mucosal healing has been known to reduce subsequent rates of relapse, hospital admission, and surgery [4, 6]. Recent findings indicate that patients whose Mayo endoscopic subscore is 0 have a better prognosis than those with a score of 1. It is therefore favorable to define mucosal healing as an endoscopic subscore of 0 (this state is referred to as “complete mucosal healing” in this article) as the desired therapeutic goal [5, 7, 8].

Active UC is basically treated with oral mesalazine preparations. In the treatment of lesions in the distal colon, local preparations including mesalazine enemas and suppositories and adrenocortical hormone enemas and suppositories are used alone or in combination with other drugs [9, 10]. Budesonide foam is a spray aerosol used for enemas, which contains the active pharmaceutical ingredient of budesonide, a synthetic glucocorticoid. Budesonide is characterized by higher receptor affinities than other glucocorticoids, e.g., by about 60 times compared with prednisolone and by 16 times compared with betamethasone. Budesonide is therefore expected to exert a potent anti-inflammatory action at the site of administration [11]. It is accepted, on the basis of its reported lower bioavailability, that budesonide is associated with less frequent systemic glucocorticoid-related adverse drug reactions than other adrenocortical hormone preparations [12, 13].

In Europe, Dr. Falk Pharma GmbH (hereinafter referred to as Dr. Falk) developed budesonide foam as a drug for achieving remission induction therapy for active UC with lesions limited to the segment from the rectum to the sigmoid colon (brand name: Budenofalk^®^ 2 mg/dose rectal foam). This drug was approved for the dosage and administration of 2 mg once daily in Great Britain in 2006, followed by approval in 36 other countries to date. In the USA, Salix Pharmaceuticals, Ltd. (hereinafter referred to as Salix) has submitted an approval application for budesonide foam aiming at clinical remission in the target population of UC patients with lesions limited to a 40-cm segment from the anus based on the standard dosage and twice-daily administration for 2 weeks, followed by once-daily dosing for 4 weeks (brand name Uceris^®^ 2 mg rectal foam).

In our exploratory phase II study in patients with distal colitis, twice-daily (BID) administration of budesonide 2-mg foam for 6 weeks induced complete mucosal healing of distal lesions more effectively than once-daily administration [14].

The effects of budesonide foam in patients with more extensive lesions, such as left-sided colitis and pancolitis, have not however been investigated. It may be critical to obtain complete mucosal healing at the distal colon to ameliorate patients’ symptoms and improve quality of life even in those with left-sided colitis and pancolitis. This phase III study examined mucosal healing achieved by BID budesonide 2-mg foam in patients with mild-to-moderate UC patients, including left-sided colitis and pancolitis, with distal active inflammation. A subset of the subjects continued the allocated treatment for a total of 12 weeks, to allow examination of the safety and efficacy of long-term treatment with budesonide foam.

## Methods

### Study design

The present trial consisted of two 6-week treatment phases. The first phase was a double-blind 6-week treatment period wherein patients were randomized at a ratio of 1:1 to receive BID budesonide 2-mg foam (2 mg/25 ml), or placebo foam. Patients were allocated to each group employing the minimization method based on the following allocation factors: use of local preparations for treatment in the current active period; the total stool frequency subscore, rectal bleeding subscore, endoscopic subscore at baseline (3–4 or 5–6), and the extent of the lesion of the underlying condition (localized between the rectum and sigmoidal colon, or extending to the adoral segment beyond the sigmoidal colon). All patients, investigators, and study sponsors were blinded until all observations, evaluations, and data collection had been completed, and the prespecified statistical analysis plans were finalized.

Continuation of study treatment was allowed up to week 12 in patients with an endoscopic subscore of 1 at week 6, for whom continuous treatment was assessed as being necessary by the attending physician.

### Patients

This multicenter, randomized, double-blind, placebo-controlled, parallel-group study was conducted in conformity with the principles of Good Clinical Practice at 45 centers in Japan between December 2014 and March 2016. This study was performed in accordance with the ethical standards laid down in the 1964 Declaration of Helsinki and its later amendments. The institutional review board of each center approved the protocol. All patients gave written informed consent prior to inclusion in this study. Eligible patients were 16 years of age or older. All were outpatients with active UC. Disease activity was assessed with a Modified Mayo Disease Activity Index (MMDAI) score. The original Mayo Index was modified, omitting friability from the endoscopic subscore definition [15].

The enrollment criteria were stool frequency subscore of 0–2, rectal bleeding subscore of 1–2, endoscopic subscore of 2 in the segment from the rectum to the sigmoid colon and 0–1 in the adoral segment beyond the sigmoid colon, and 12 weeks or longer since the diagnosis of UC. Oral 5-aminosalicylic acid (5-ASA) agents, or oral salazosulfapyridine agents, or probiotics were permitted at stable doses as concomitant therapies. Use of the following drugs and therapies was prohibited during this study: rectal preparations or suppositories of 5-ASA, suppositories of salazosulfapyridine, corticosteroid preparations, cytapheresis, immunomodulators, anti-tumor necrosis factor antibody preparations, and surgical treatment for UC. Patients were excluded from enrollment in this study if they had any of the following: a history of colon resection, irritable bowel syndrome, intolerance or allergic reaction to budesonide, or a plasma cortisol level below 6.2 μg/dl.

### Efficacy evaluations

Patients were evaluated at weeks 0 (baseline), 2, 4, and 6, or at the withdrawal visit. The MMDAI score was assessed by colonoscopy and data recorded in a symptom diary, based on the 3 days closest to each visit. Endoscopic examination was performed by total colonoscopy at week 0 and total colonoscopy or sigmoidoscopy at week 6 or the withdrawal visit. The endoscopic subscoring by the central committee was applied for analysis of the data. All evaluations were conducted in a blinded manner. The primary endpoint was the percentage of complete mucosal healing of distal lesions, defined as the percentage of patients with an endoscopic subscore of 0, at week 6. The evaluation was based on the lesion in the segment between the rectum and sigmoidal colon. The secondary endpoint was the clinical remission percentage, defined as the percentage of patients with a rectal bleeding subscore of 0, endoscopic subscore of 0 or 1, and stool frequency subscore of 0 or a decrease in this subscore by at least 1 from baseline.

When the allocated intervention was continued, patients were evaluated at weeks 8, 10, and 12, or at the withdrawal visit. The MMDAI score was assessed by colonoscopy and the data from the symptom diary, based on the 3 days closest to each visit. Endoscopic findings were obtained with total colonoscopy or sigmoidoscopy at week 12 or at the withdrawal visit.

### Safety and acceptance evaluations

Patients underwent measurement of vital signs (blood pressure, pulse rate, and body temperature), assessment of adverse effects, review of concomitant therapies, hematologic and blood biochemistry tests, and urinalysis. Patients were weighed, and glycosylated hemoglobin (HbA1C) was measured at baseline and week 6 and, in patients continuing the treatment, at week 12. Plasma concentrations of cortisol and adrenocorticotropic hormone (ACTH) were measured at baseline, week 6, and follow-up (at week 12 in patients who continued the treatment). If an adverse event had not resolved by week 6 or 12 or the withdrawal visit, follow-up was continued until the event had fully resolved. To assess patients’ acceptance at week 6 or the withdrawal visit, all patients were asked to complete questionnaires on general problems related to handling of the device, difficulty of administration, retention and so on.

### Sample size

We referred to the results of a phase II study of budesonide foam in Japanese active UC patients [14]. The percentage of complete mucosal healing was 46.4% in the budesonide foam BID group. We took a conservative approach of determining the target sample size of the present study based on the one-sided 95% confidence interval of the percentage of complete mucosal healing. Based on the Wald method, the upper limit of the one-sided 95% confidence interval of the percentage of complete mucosal healing was 35.5% in the BID group and 10.7% in the placebo group. Assuming that the percentage of complete mucosal healing of the general population is 35.5% in the BID group and 10.7% in the placebo group, the required sample size was 59 patients/group at a significance level of 0.05 (two-sided) and power of 90%. Estimating that one patient would drop out of the study, we aimed to include 60 patients in each group, i.e., 120 patients in total.

### Statistical methods

The demographics and baseline characteristics of patients were summarized to assess the balance between the two treatment groups employing descriptive statistics in the full analysis set (FAS) and per protocol set (PPS). The FAS consisted of all patients who were enrolled, randomized, received at least one dose of study treatment, and had at least one available efficacy data point. The PPS consisted of the FAS after exclusion of patients who did not satisfy any of the inclusion criterion or met any of the exclusion criterion, patients who were treated with a study drug with a different drug number from the allocated number, those who used prohibited drugs, patients whose treatment compliance was poor, those who were lost to follow-up, and patients with missing data. The safety analysis set consisted of all patients who were enrolled, randomized, and received at least one dose of study treatment. The efficacy analysis was performed for the FAS. In addition, the sensitivity analysis was conducted for the PPS. The safety analysis was performed for the safety analysis set. Data from patients who continued study treatments for 12 weeks were analyzed separately.

For the efficacy analysis of the primary endpoint, the superiority test was performed to compare the percentage of complete mucosal healing at week 6 between the budesonide foam group and the placebo group by applying a logistic regression model with the following factors as covariates: use of local preparations for UC treatment in the current active phase (not used or used); sum of the stool frequency, rectal bleeding, and endoscopic subscores at baseline (≤4 or ≥5); and extent of the lesion (limited to sigmoid colon from rectum or not).

In post hoc analyses, the percentages of complete mucosal healing and clinical remission were evaluated employing the *χ* ^2^ test in the following seven patient subgroups: patients with left-sided colitis plus pancolitis, proctitis alone, left-sided colitis alone, pancolitis alone; previous use of 5-ASA (no or yes), dose of oral 5-ASA (low or high), and baseline MMDAI score (≤5 or ≥6). The high dose of 5-ASA was defined as 4.0 g of Pentasa^®^ or 3.6 g of Asacol^®^ or 4.0 g of salazosulfapyridine. The low dose of 5-ASA was defined as less than the above doses or no use of oral 5-ASA. All statistical analyses were performed at a significance level of 0.05 (two-sided).

## Results

### Patient disposition, baseline demographics, and clinical characteristics

**Fig. 1:**
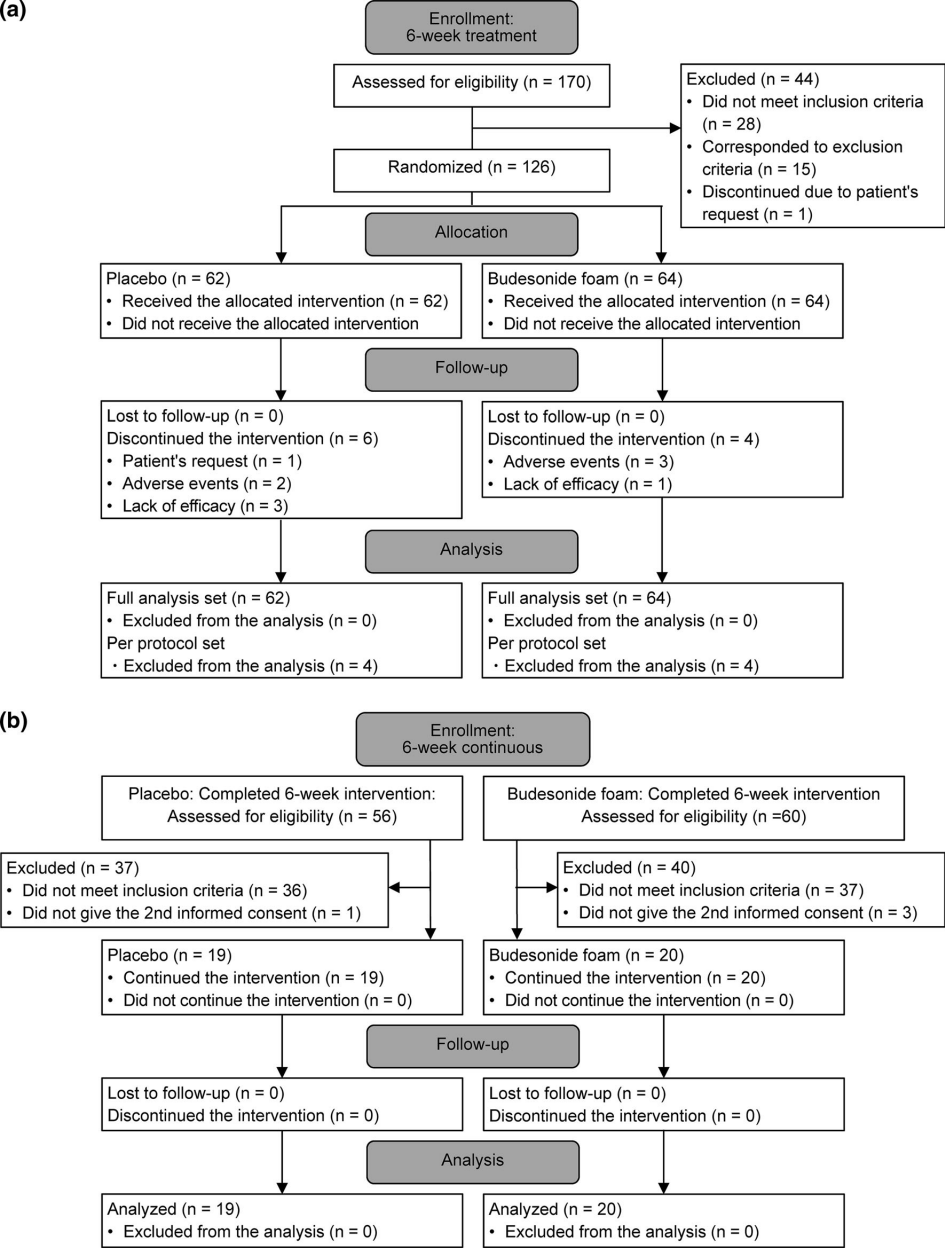
Patient flow diagram. **a** Initial 6-week treatment (weeks 1–6), **b** further 6-week continuous treatment (weeks 7–12)

Figure 1 shows the disposition of patients. Among 170 patients who were assessed for eligibility, 44 were excluded from this study. The remaining 126 patients were randomized to the 6-week double-blind treatment phase: 116 patients completed the study treatments. The FAS for the efficacy and safety evaluations included all 126 patients. There were no substantial differences in baseline demographics or clinical characteristics between the two groups of patients (Table 1). Clinical manifestations of the 126 patients enrolled in this study were 16 with pancolitis, 65 with left-sided colitis, and 45 with proctitis.

**Table 1:** Baseline demographics and clinical characteristics

	Placebo (*n* = 62)	Budesonide foam (*n* = 64)	Total (*n* = 126)
Age (years), mean (SD)	41.9 (12.3)	40.2 (12.2)	41.0 (12.2)
Male sex, *n* (%)	31 (50.0)	32 (50.0)	63 (50.0)
Body weight (kg), mean (SD)	61.3 (12.7)	57.1 (11.6)	59.2 (12.3)
Current smoker, *n* (%)	6 (9.7)	0 (0.0)	6 (4.8)
Duration of disease (years), *n* (%)	3.5 (3.5)	5.7 (5.6)	5.8 (5.9)
<5	33 (53.2)	32 (50.0)	65 (51.6)
≥5	29 (46.8)	32 (50.0)	61 (48.4)
Clinical course, *n* (%)			
First attack	1 (1.6)	4 (6.3)	5 (4.0)
Relapsing/remitting	61 (98.4)	60 (93.8)	121 (96.0)
Duration of present active phase *n* (%)			
<4 weeks	32 (51.6)	26 (40.6)	58 (46.0)
≥4 weeks	30 (48.4)	38 (59.4)	68 (54.0)
Extent of past lesions, *n* (%)			
Pancolitis	5 (8.1)	11 (17.2)	16 (12.7)
Left-sided colitis	34 (54.8)	31 (48.4)	65 (51.6)
Proctitis	23 (37.1)	22 (34.4)	45 (35.7)
MMDAI, *n* (%)			
Scores 3–5	19 (30.6)	21 (32.8)	40 (31.7)
Scores 6–9	43 (69.4)	43 (67.2)	86 (68.3)
Endoscopic subscore assessed by central committee			
1	10 (16.1)	12 (18.8)	22 (17.5)
2	46 (74.2)	46 (71.9)	92 (73.0)
3	6 (9.7)	6 (9.4)	12 (9.5)
Previous medication for UC, *n* (%)			
Oral 5-ASA high dose	42 (67.7)	44 (68.8)	86 (68.3)
Oral 5-ASA low dose	20 (32.2)	20 (31.2)	40 (31.7)
5-ASA enema or suppository	28 (45.2)	33 (51.6)	61 (48.4)

*SD* standard deviation, *MMDAI* Modified Mayo Disease Activity Index

### Efficacy

#### Complete mucosal healing of distal lesions and clinical remission in UC patients and the subgroups of patients with proctitis, left-sided colitis, and pancolitis

**Fig. 2:**
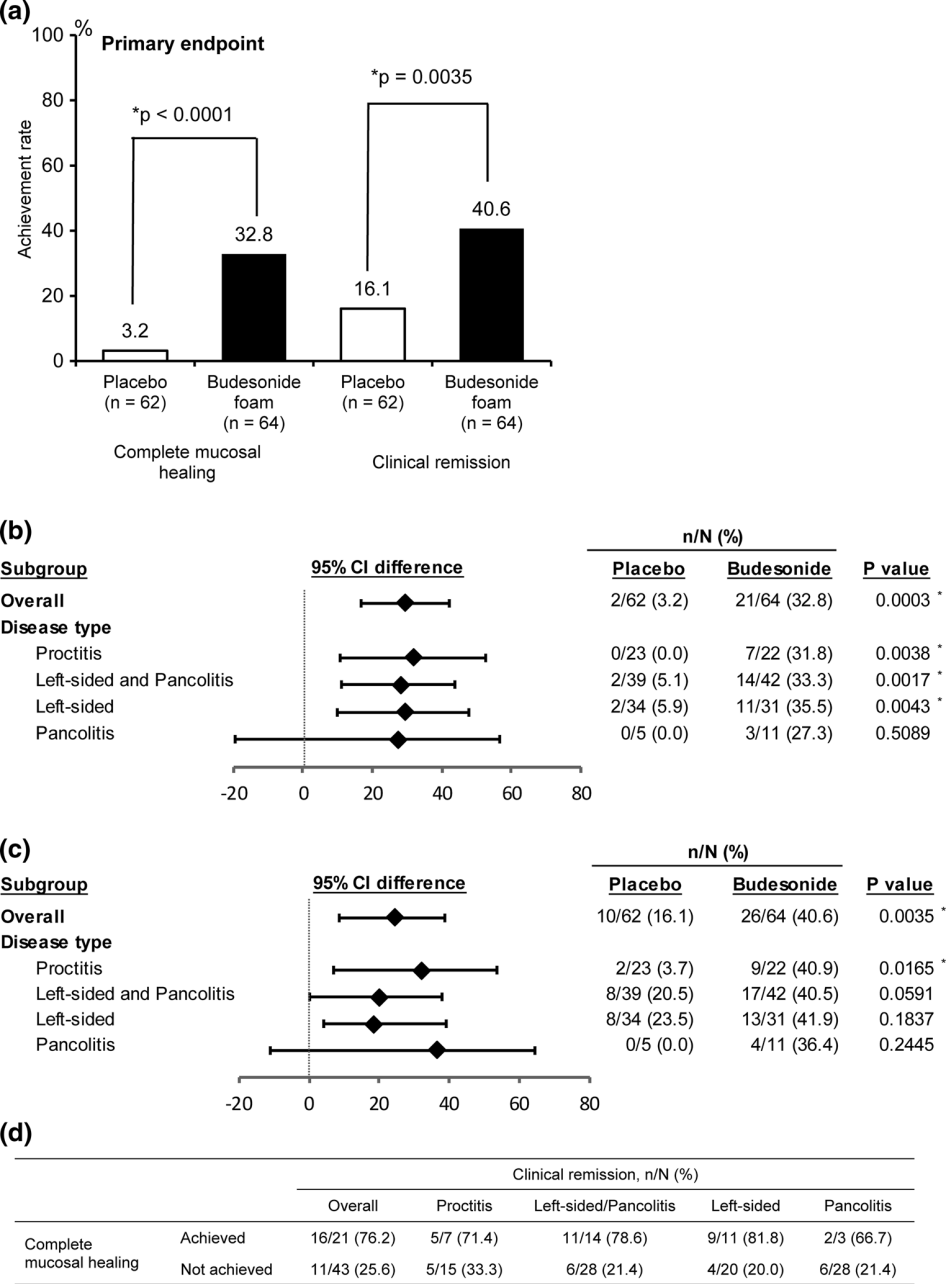
**a** Complete mucosal healing of distal lesions and clinical remission in patients with ulcerative colitis at week 6. **b** Complete mucosal healing of distal lesions and **c** clinical remission in subgroups of patients with proctitis, left-sided colitis, and pancolitis at week 6. **d** Clinical remission in patients who achieved complete mucosal healing of distal lesions in the budesonide group at week 6. Statistical analyses were performed at a significance level of 0.05 (two-sided). *Asterisks* significant difference, *CI* confidence interval

Results of this clinical study confirmed the efficacy of budesonide foam for the primary endpoint (Fig. 2a). Specifically, the percentages of both complete mucosal healing of distal lesions and clinical remission were significantly higher in the budesonide foam group than in the placebo group (Fig. 2a). Subgroup analysis showed that budesonide foam was also effective for complete mucosal healing of distal lesions in patients with left-sided colitis and pancolitis (Fig. 2b). The clinical remission percentage also showed improvement even in patients with left-sided colitis and pancolitis, although the difference did not reach statistical significance (Fig. 2c). Among patients with proctitis as well as those with left-sided colitis and pancolitis in the budesonide foam group, the clinical remission percentage tended to be higher in those who achieved complete mucosal healing of distal lesions than in patients who did not reach this target (Fig. 2d). This result indicated complete mucosal healing of distal lesions to promote clinical remission of mild-to-moderate UC patients with distal active inflammation.

#### Other subgroup analyses

**Fig. 3:**
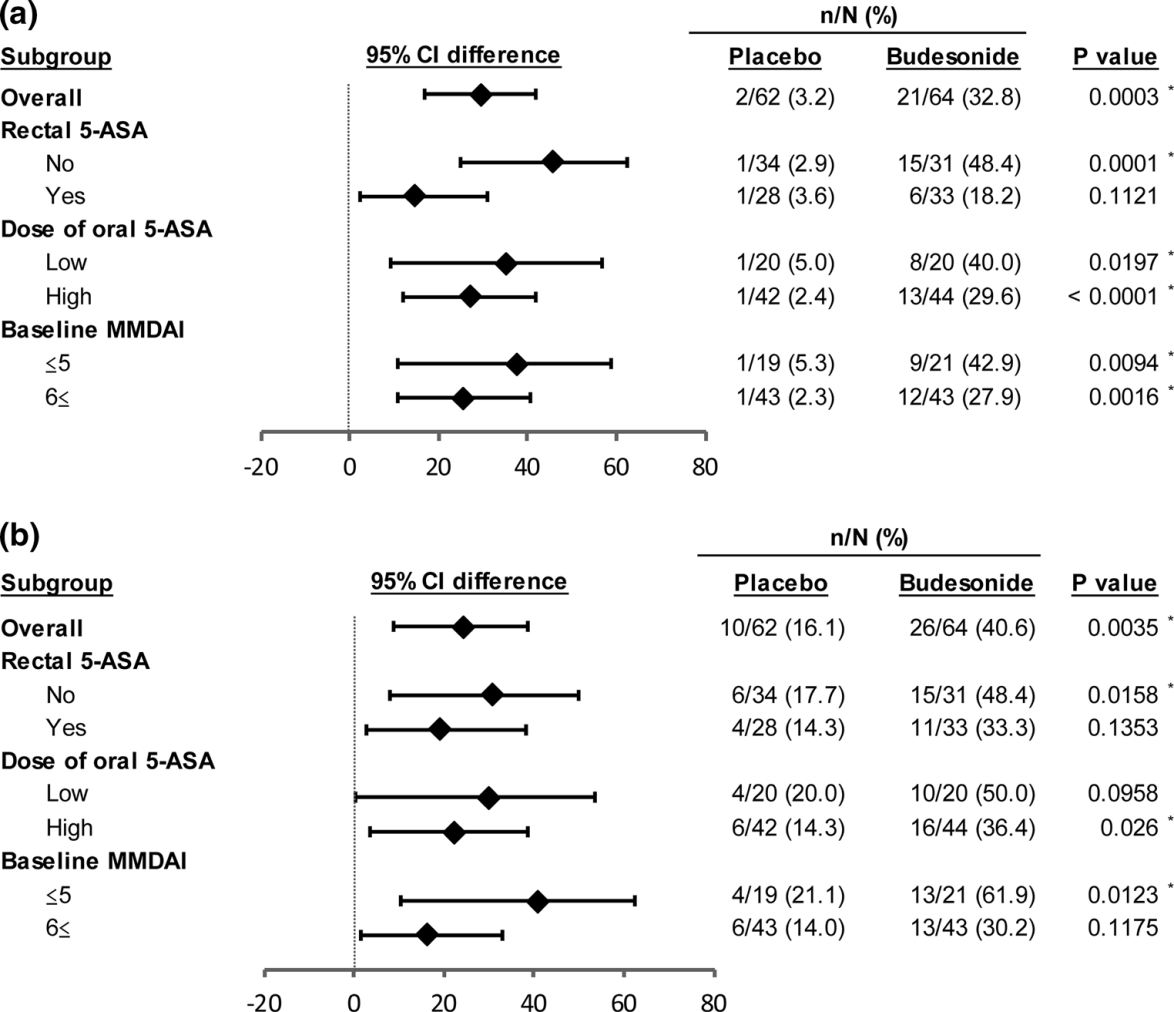
Other subgroup analysis. **a** Complete mucosal healing of distal lesions. **b** Clinical remission. Statistical analyses were performed at a significance level of 0.05 (two-sided). High-dose 5-ASA means 4.0 g of Pentasa^®^ or 3.6 g of Asacol^®^ or 4.0 g of salazosulfapyridine. The low dose means less than these dosages or no use of oral 5-ASA. *Asterisks* significant difference, *5-ASA* 5-aminosalicylic acid, *CI* confidence interval, *MMDAI* Modified Mayo Disease Activity Index

Figure 3 shows the results of the other subgroup analyses. Budesonide foam was generally more effective than the placebo in all subgroups. Importantly, the efficacy of budesonide foam on mucosal healing of distal lesions and clinical remission was confirmed in patients who had been treated with high doses of oral 5-ASA (Fig. 3a, b). None of the following factors was found to exert effects: duration of remission induction therapy in the current active phase, sex, and disease duration (data not shown).

#### Other endpoints

**Fig. 4:**
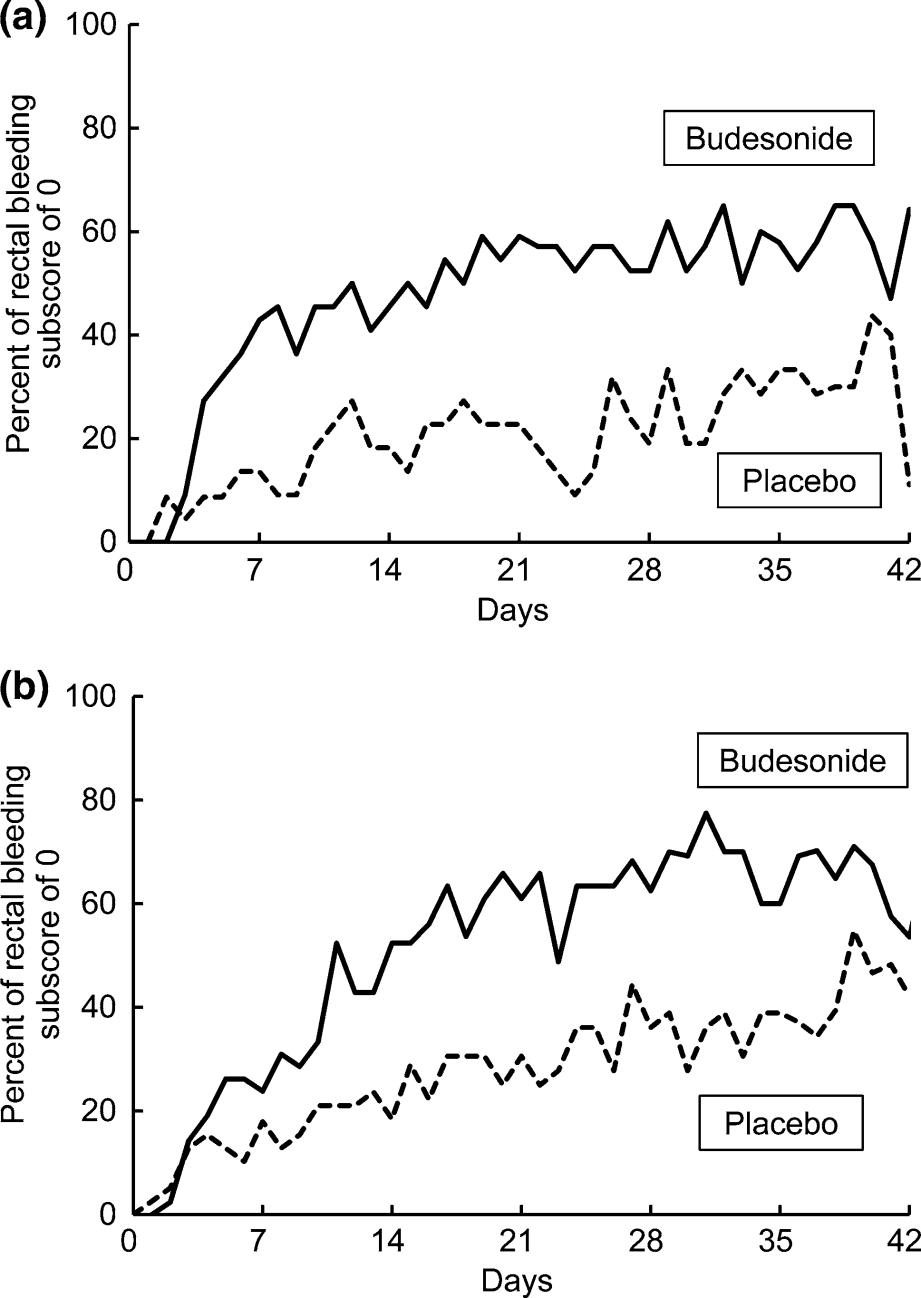
Percentage of patients with a rectal bleeding subscore of 0 on each day. **a** Proctitis subgroup. **b** Left-sided colitis and pancolitis subgroups

Table S1 shows the results of the efficacy analysis for other endpoints. The percentage of patients with an endoscopic subscore of at most 1 was significantly higher in the budesonide foam group than in the placebo group (75.0% versus 35.5%, *p* < 0.0001). The percentage of patients with an MMDAI score of at most 1 was significantly higher in the budesonide foam group than in the placebo group (34.4% versus 4.8%, *p* = 0.0002). Table S1 also shows the percentage of patients whose rectal bleeding subscore was 0 at weeks 2, 4, and 6. In the budesonide foam group, the percentage of patients with elimination of rectal bleeding was significantly higher at all time points than in the placebo group (53.1%, 65.6%, and 68.3% versus 23.7%, 32.2%, and 42.9%, *p* = 0.0015, 0.0003, and 0.0053, respectively). As shown in Fig. 4, rectal bleeding disappeared within several days after the start of treatment with budesonide foam in the left-sided colitis and pancolitis subgroups as well as in the proctitis subgroup.

### Safety

#### Summary of adverse events

Table 2 summarizes adverse events. There were no deaths during the study period. The safety of the study drug in patients treated for 6 weeks was assessed using the safety analysis set that included all 126 randomized patients. The incidence of adverse events was 45.3% in the budesonide foam group and 40.3% in the placebo group. The incidence of study drug-related adverse events was 17.2% in the budesonide group and 9.7% in the placebo group. Serious adverse events were thyroid microcarcinoma in one patient in the budesonide foam group and hospitalization due to asthma in one patient in the placebo group. The thyroid microcarcinoma was found 20 days after the start of study treatment on the basis of body weight loss. This female patient had herself noticed the weight loss before treatment initiation. Because it was unlikely that thyroid cancer would have developed within 20 days after the start of study treatment, the investigator assessed this event as being unlikely to be related to the administration of budesonide foam. A relationship with the study drug was also ruled out for the patient receiving the placebo. The incidence of adverse events leading to treatment discontinuation was 6.3% in the budesonide foam group and 3.2% in the placebo group.

**Table 2:** Adverse events

	From week 0 to week 6		From week 6 to week 12^c^	
	Placebo (*n* = 62)	Budesonide foam (*n* = 64)	Placebo (*n* = 19)	Budesonide foam (*n* = 20)
Summary of adverse events, *n* (%)				
Adverse events	25 (40.3)	29 (45.3)	4 (21.0)	6 (30.0)
Study drug-related adverse events	6 (9.7)	11 (17.2)	2 (10.5)	1 (5.0)
Death	0 (0.0)	0 (0.0)	0 (0.0)	0 (0.0)
Serious adverse events	1 (1.6)	1 (1.6)	0 (0.0)	0 (0.0)
Adverse events leading to treatment discontinuation	2 (3.2)	4 (6.3)	0 (0.0)	0 (0.0)
Common adverse events, *n* (%)^a^				
Infections and infestations				
Nasopharyngitis	3 (4.8)	7 (10.9)	0 (0.0)	1 (5.3)
Upper respiratory tract infection	0 (0.0)	0 (0.0)	1 (5.3)	0 (0.0)
Tinea pedis	0 (0.0)	0 (0.0)	0 (0.0)	1 (5.3)
Nervous system disorders				
Headache	3 (4.8)	2 (3.1)	0 (0.0)	0 (0.0)
Vascular disorders				
Hypertension	1 (1.6)	3 (4.7)	0 (0.0)	0 (0.0)
Respiratory, thoracic and mediastinal disorders				
Upper respiratory tract inflammation	7 (11.3)	1 (1.6)	1 (5.3)	0 (0.0)
Asthma	2 (3.2)	0 (0.0)	0 (0.0)	0 (0.0)
Gastrointestinal disorders				
Vomiting	0 (0.0)	2 (3.1)	0 (0.0)	0 (0.0)
Gastric ulcer	0 (0.0)	0 (0.0)	1 (5.3)	0 (0.0)
Stomatitis	0 (0.0)	0 (0.0)	0 (0.0)	1 (5.3)
Frequent bowel movements	0 (0.0)	0 (0.0)	0 (0.0)	1 (5.3)
Skin and subcutaneous tissue disorders				
Eczema	0 (0.0)	1 (1.6)	1 (5.3)	0 (0.0)
Rash	0 (0.0)	1 (1.6)	0 (0.0)	1 (5.3)
General disorders and administration site conditions				
Pyrexia	0 (0.0)	2 (3.1)	1 (5.3)	1 (5.3)
Investigations^b^				
Blood creatine phosphokinase increased	0 (0.0)	2 (3.1)	0 (0.0)	0 (0.0)
Blood uric acid increased	0 (0.0)	0 (0.0)	0 (0.0)	1 (5.3)

^a^Defined as an adverse event that occurred in at least 2% of patients in any group

^b^Except plasma cortisol or plasma ACTH decrease

^c^Adverse events newly developing during the period from week 6 to week 12

In patients who were continuously treated for 12 weeks, the safety analysis of the study drug included 39 patients. From week 6 to week 12, the incidence of adverse events was 30.0% in the budesonide foam group and 21.0% in the placebo group. The incidence of study drug-related adverse events was 5.0% in the budesonide group and 10.5% in the placebo group. None of the patients who continued study treatment for 12 weeks experienced any serious adverse events or adverse events leading to treatment discontinuation in either group.

In patients who continued study treatment up to week 12, the incidence of adverse events did not increase, nor was the onset of any serious adverse reactions observed, from week 6 through week 12. The overall adverse event incidences did not differ between the budesonide foam and placebo groups.

#### Glucocorticoid-related adverse events

Glucocorticoid-related adverse events occurred in five patients in the budesonide foam group and three patients in the placebo group, i.e., the incidence was not higher in the active drug group. The glucocorticoid-related adverse events in the budesonide foam group were insomnia in one patient, hypertension in three, and peripheral edema in three. Among those who continued study treatment for 12 weeks, gastric ulcer occurred in one patient in the placebo group. No glucocorticoid-related adverse events were reported in the budesonide foam group (Table 2; data not shown).

Decreased plasma concentrations of cortisol and ACTH were found in about half of patients at week 6 and week 12 in the budesonide foam group. Follow-up test values were normal in all of these patients at week 6 (Table 3). Among those who continued study treatment up to week 12, concentrations of plasma cortisol and plasma ACTH were still below the normal ranges at follow-up in two patients (Table 3). The test values were close to the normal ranges, with no onset of any noteworthy adverse symptoms in either patient. The mean plasma concentrations of cortisol and ACTH showed substantial reductions at week 6, and had returned to normal levels at the final follow-up visit (Fig. S1a; data for ACTH, for which similar results were obtained, not shown). In patients who continued study treatment up to week 12, both of these plasma concentrations were also reduced at week 12, but had returned to normal levels at follow-up 12 weeks later (Fig. S1b; data for ACTH, for which similar results were obtained, not shown).

**Table 3:** Decreases in morning plasma cortisol and ACTH

	Placebo (*N* = 62)	Budesonide foam (*N* = 64)
Decreases in morning plasma cortisol^a^, *n*/*N* ^b^ (%)		
Baseline	0/62 (0.0)	0/64 (0.0)
Week 6	2/51 (3.9)	30/59 (50.8)
Week 12	1/19 (5.3)	11/19 (57.9)
Follow-up		
After 6-week treatment	1/30 (3.3)	0/36 (0.0)
After 12-week treatment	0/17 (3.2)	2/20 (10.0)
Decreases in morning plasma ACTH^c^, *n*/*N* ^b^ (%)		
Baseline	3/62 (4.8)	5/63 (7.9)
Week 6	1/51 (2.0)	18/59 (30.5)
Week 12	0/19 (0.0)	8/19 (42.1)
Follow-up		
After 6-week treatment	2/30 (6.7)	0/36 (0.0)
After 12-week treatment	0/17 (0.0)	2/20 (10.0)

*ACTH* adrenocorticotropic hormone

^a^Defined as plasma cortisol concentration <6.2 μg/dl

^b^Denominator *N* is the number of patients with a measured value in each given week during this study

^c^Defined as plasma ACTH concentration <7.2 pg/dl

### Patient acceptance

All of the 126 patients who were treated with the study drug responded to the questionnaires. Figure S2 shows the results. Experience using other enemas or suppositories was reported by 87% of the patients. About 80% of the patients responded that it was “easy” or “very easy” to handle the device and perform the application procedure in the standing posture, and “none” had experienced a retention problem. The time required for application was less than 3 min in 60% of patients, and within 5 min in 90%. These results indicated that administration of budesonide foam was less stressful for patients than the existing enemas for which patients had to lie down and remain at rest for a certain period of time to achieve appropriate application.

## Discussion

This was a phase III clinical study involving patients with mild-to-moderate UC, aiming to confirm for the first time that BID budesonide foam, administered at a 2-mg dose, induced complete mucosal healing of distal lesions in UC patients including those with left-sided colitis and pancolitis. Furthermore, our results showed that complete mucosal healing of distal lesions promotes clinical remission in patients with left-sided colitis and pancolitis as well as in those with proctitis. Thus, budesonide foam, which induced complete mucosal healing of distal lesions, is anticipated to be an effective therapeutic option not only for patients with proctitis but also those with left-sided colitis and pancolitis who have distal active inflammation.

On the basis of a previous study showing that budesonide foam is a drug that reaches as far as the sigmoidal colon [16], this drug is indicated in Europe for proctitis and sigmoiditis, and in the USA for patients with inflammatory lesions localized within a 40-cm segment from the anus. Additional local preparations are used in combination with oral preparations for the treatment of distal lesions that are not improved by oral therapies in patients with left-sided colitis or pancolitis [9, 10]. There are some UC cases whose lesions develop from the rectum or are particularly severe in the rectum. Amelioration of distal inflammation therefore probably results in improvement of clinical symptoms. It has in fact been reported that in patients with pancolitis and those with left-sided colitis, use of 5-ASA suppositories is effective not only for ameliorating mucosal lesions but also for induction of clinical remission [17]. This confirmatory study showed that budesonide foam, given twice daily for 6 weeks, induced complete mucosal healing of distal lesions in 32.8% of patients (Fig. 2a). This drug also induced complete mucosal healing of distal lesions as well as clinical remission at a similar frequency in patients with left-sided colitis and in patients with pancolitis. In our study, the percentages of complete mucosal healing of distal lesions and clinical remission were 31.8% and 40.9% in the proctitis subgroup, and 33.3% and 40.5% in the left-sided colitis and pancolitis subgroup, respectively, showing budesonide foam to be effective for mild-to-moderate UC regardless of subtype (Fig. 2b, c). Rectal bleeding disappeared within several days after the start of treatment with budesonide foam in the left-sided colitis and pancolitis subgroup as well as in the proctitis subgroup (Fig. 4). Among patients who achieved complete mucosal healing of distal lesions, more attained clinical remission as compared with the patients who did not reach this goal (Fig. 2d). These results indicated that complete mucosal healing of distal lesions results in the improvement of systemic clinical symptoms in UC patients.

A reported study compared patients who were treated with once-daily budesonide 2-mg enema and patients given prednisolone 31.25-mg enema for 8 weeks. While the percentage of clinical remission with non-inflamed mucosa in the budesonide enema group was similar to that in patients receiving prednisolone enemas (36% vs. 47%), morning plasma cortisol levels were significantly suppressed only in the prednisolone group [18]. From these results, the authors concluded that budesonide may be preferable to prednisolone enema since it causes fewer systemic effects. Our study showed BID budesonide foam for 12 weeks to be well tolerated, although plasma cortisol suppression was observed in some patients, suggesting that budesonide foam can be used without major safety concerns, as reported previously. Lémann and colleagues reported a 4-week comparative study of the budesonide 2-mg enema versus a 5-ASA 1-g enema. Their results demonstrated the budesonide and 5-ASA enemas to be similarly tolerated, but the clinical remission percentage in the 5-ASA treatment group was significantly higher than that in the budesonide treatment group (60% vs. 38%) [19]. The European Crohn’s and Colitis Organization (ECCO) guideline states that the efficacy of topical 5-ASA is superior to that of topical corticosteroid and their research results were consistent with this statement. The ECCO guideline also describes a clinical benefit of corticosteroids which have shown efficacy in patients with 5-ASA failure [10]. In our present study, in the subgroup that had received previous treatment with rectal 5-ASA, 18% of patients achieved complete mucosal healing of distal lesions and 33.3% achieved clinical remission. This result may indicate the potential efficacy of budesonide foam in patients with 5-ASA failure.

While enema therapy is often associated with poor compliance, foam preparations are less stressful for patients, because they are easier to administer with fewer concerns over leakage after application than with other types of enemas [20]. In our phase II study, many patients responded that they had experienced no difficulty in application and that the foam preparation was acceptably easy to use even when it was administered twice daily [14]. In the present clinical study, 87% of the patients had prior experience with using other enemas or suppositories. Similarly, BID budesonide foam was not particularly stressful for approximately 80% of the patients. Thus, budesonide foam is thought to be far superior to other enema preparations in terms of achieving good compliance.

On the basis of the subgroup analysis, budesonide foam and the placebo showed similar efficacies in all subgroups, according to the oral dose of 5-ASA or baseline MMDAI score. The lack of significant differences among the subgroups was thought to be due to insufficient sample size. Clinical remission and complete mucosal healing of distal lesions were obtained in approximately one-third of the patients receiving high doses of oral 5-ASA. This result indicates that budesonide foam also exerts therapeutic efficacy even in high-dose 5-ASA refractory UC patients. Consistently, Bosworth et al. [21] have confirmed budesonide foam to be effective in patients with proctitis and sigmoiditis using oral 5-ASA. Sandborn et al. [22] reported that budesonide foam was superior to placebo regardless of whether disease severity was mild or moderate. Our results are consistent with these earlier reports.

Several research teams have reported the effect of complete mucosal healing with additional use of local 5-ASA in UC patients who had used low-dose oral 5-ASA. In a study by Sandborn et al., examining the effects of a combination of oral 5-ASA at 2.4 g/day and an enema with 5-ASA suspension at 4 g/day, the percentage of patients who achieved a mucosal subscore of 0 was 22.7% in those who used only oral 5-ASA and 25.0% in those given the combination regimen with the 5-ASA enema [23]. In a study by Kobayashi et al., who combined oral 5-ASA at 2.4 g/day or lower with an additional 5-ASA suppository once-daily dose of 1 g for 4 weeks, the complete mucosal healing percentage was 29.0% (19 of 64 patients) [24]. In our study, wherein BID budesonide foam was added to the treatment regimens of patients who had been using low-dose oral 5-ASA as in the above previous studies, the complete mucosal healing percentage was 40.0%, indicating treatment with BID budesonide foam to be very useful in the initial management of UC before dose escalation of 5-ASA. A previous study showed that complete mucosal healing resulted in a subsequent lower recurrence rate and more favorable prognosis [25]. It is advantageous in terms of both quality of life for patients and healthcare economics to achieve complete mucosal healing in the early stage of treatment, because maintenance of remission with less use of additional treatments can thereby be expected.

None of our study results for BID budesonide foam raised any safety concerns. During the 6-week treatment period, there was no substantial difference in the onset of glucocorticoid-related adverse reactions as compared with a previous study [26]. Decreased plasma cortisol levels had recovered to within the normal range at the follow-up visit after the last dose in both patients treated for 6 weeks and those treated for 12 weeks. None of patients who were treated for 12 weeks experienced any previously unknown adverse events and the incidence of adverse events was not increased. It was found to be safe to use budesonide foam during the entire study period.

In this study, it was not mandatory to perform endoscopy at week 12. However, 13 patients in the placebo group and 15 in the budesonide foam group underwent this procedure at this time point. None of the 13 placebo patients achieved complete mucosal healing. Among the 15 patients in the budesonide foam group, complete mucosal healing was confirmed in 6 (40.0%). None of these six patients had achieved this target at week 6 (data not shown). Although further studies are needed, prolonged treatment through week 12 may improve the complete mucosal healing percentage achieved with budesonide foam in patients who did not obtain mucosal healing at week 6.

Our study has limitations. The sample size of the left-sided colitis and pancolitis subgroup was relatively small. Pancolitis and left-sided UC patients with severe inflammation in the adoral segment beyond the sigmoid colon or without active inflammation in distal lesions were excluded from this study. Our results are thus not applicable to such patients. However, our study focused on patients with mild-to-moderate UC, not requiring biologics, immunomodulators, or hospitalization. These UC patients do not always have widespread severe inflammation, even if they have pancolitis. In fact, of 170 potential subjects who were assessed for eligibility, only five were excluded because their endoscopic subscores in the adoral segment beyond the sigmoid colon were 2 or 3. Thus, although restricted to mild-to-moderate UC patients with distal active inflammation, our study has generalizability. On the other hand, because we did not apply limitations on the type of disease or the concomitant use of oral 5-ASA or its dosage, our results are widely applicable to patients with mild-to-moderate UC, regardless of these features.

Results of our study confirmed BID budesonide 2-mg foam to be an effective and safe drug for ameliorating the clinical symptoms of patients with mild-to-moderate UC including those with left-sided colitis and pancolitis, by inducing complete mucosal healing of distal lesions.

## Electronic supplementary material

Below is the link to the electronic supplementary material.

Supplementary material 1 (PDF 378 kb)

## References

[1] Langholz E, Munkholm P, Davidsen M (1996). Changes in extent of ulcerative colitis: a study on the course and prognostic factors. Scand J Gastroenterol.

[2] Ayres RC, Gillen CD, Walmsley RS (1996). Progression of ulcerative proctosigmoiditis: incidence and factors influencing progression. Eur J Gastroenterol Hpatol.

[3] Henriksen M, Jahnsen J, Lygren I (2006). Ulcerative colitis and clinical course: results of a 5-year population-based follow-up study (the IBSEN study). Inflamm Bowl Dis.

[4] Walsh A, Palmer R, Travis S (2014). Mucosal healing as a target of therapy for colonic inflammatory bowel disease and methods to score disease activity. Gastrointest Endosc Clin N Am.

[5] Mazzuoli S, Guglielmi FW, Antonelli E (2013). Definition and evaluation of mucosal healing in clinical practice. Dig Liver Dis.

[6] Colombel JF, Rutgeerts P, Reinisch W (2011). Early mucosal healing with infliximab is associated with improved long-term clinical outcomes in ulcerative colitis. Gastroenterology.

[7] Nakarai A, Kato J, Hiraoka S (2014). Prognosis of ulcerative colitis differs between patients with complete and partial mucosal healing, which can be predicted from the platelet count. World J Gastroenterol.

[8] Yokoyama K, Kobayashi K, Mukae M (2013). Clinical study of the relation between mucosal healing and long-term outcomes in ulcerative colitis. Gastroenterol Res Pract.

[9] Watanabe M, Treatment Guideline for Ulcerative Colitis (revised in fiscal year 2010) (2010). Health and labour sciences research grants from the Japanese Ministry of Health, Labour and Welfare for research on measures for intractable diseases (inflammatory bowel disease). Integrated Shared Res Rep (2010).

[10] Dignass A, Lindsay JO, Sturm A (2012). Second European evidence-based consensus on the diagnosis and management of ulcerative colitis part 2: current management. J Crohns Colitis.

[11] Brattsand R (1990). Overview of newer glucocorticosteroid preparations for inflammatory bowel disease. Can J Gastroenterol.

[12] Jönsson G, Aström A, Andersson P (1995). Budesonide is metabolized by cytochrome P450 3A (CYP3A) enzymes in human liver. Drug Metab Dispos.

[13] Ryrfeldt A, Andersson P, Edsbäcker S (1982). Pharmacokinetics and metabolism of budesonide, a selective glucocorticoid. Eur J Respir Dis Suppl.

[14] Naganuma M, Aoyama N, Suzuki Y (2016). Twice-daily budesonide 2-mg foam induces complete mucosal healing in patients with distal ulcerative colitis. J Crohns Colitis.

[15] Sandborn WJ, Kamm MA, Lichtenstein GR (2007). MMX Multi Matrix System mesalazine for the induction of remission in patients with mild-to-moderate ulcerative colitis: a combined analysis of two randomized, double blind, placebo controlled trails. Aliment Pharmacol Ther.

[16] Brunner M, Vogelsang H, Greinwald R (2005). Colonic spread and serum pharmacokinetics of budesonide foam in patients with mildly to moderately active ulcerative colitis. Aliment Pharmacol Ther.

[17] Watanabe M, Nishino H, Sameshima Y (2013). Randomised clinical trial: evaluation of the efficacy of mesalazine (mesalamine) suppositories in patients with ulcerative colitis and active rectal inflammation—a placebo-controlled study. Aliment Pharmacol Ther.

[18] Löfberg R, Ostergaard Thomsen O, Langholz E (1994). Budesonide versus prednisolone retention enemas in active distal ulcerative colitis. Aliment Pharmacol Ther.

[19] Lémann M, Galian A, Rutgeerts P (1995). Comparison of budesonide and 5-aminosalicylic acid enemas in active distal ulcerative colitis. Aliment Pharmacol Ther.

[20] Gross V, Bar-Meir S, Lavy A (2006). Budesonide foam versus budesonide enema in active ulcerative proctitis and proctosigmoiditis. Aliment Pharmacol Ther.

[21] Bosworth BP, Sandborn WJ, Rubin DT (2016). Baseline oral 5-ASA use and efficacy and safety of budesonide foam in patients with ulcerative proctitis and ulcerative proctosigmoiditis: analysis of 2 phase 3 studies. Inflamm Bowel Dis.

[22] Sandborn WJ, Bosworth B, Zakko S (2015). Budesonide foam induces remission in patients with mild to moderate ulcerative proctitis and ulcerative proctosigmoiditis. Gastroenterology.

[23] Sandborn WJ, Hanauer S, Lichtenstein GR (2011). Early symptomatic response and mucosal healing with mesalazine rectal suspension therapy in active distal ulcerative colitis—additional results from two controlled studies. Aliment Pharmacol Ther.

[24] Kobayashi K, Hirai F, Naganuma M (2014). A randomized clinical trial of mesalazine suppository: the usefulness and problems of central review of evaluations of colonic mucosal findings. J Crohns Colitis.

[25] Arai M, Naganuma M, Sugimoto S (2016). The ulcerative colitis endoscopic index of severity is useful to predict medium- to long-term prognosis in ulcerative colitis patients with clinical remission. J Crohns Colitis.

[26] Rubin DT, Sandborn WJ, Bosworth B (2015). Budesonide foam has a favorable safety profile for inducing remission in mild-to-moderate ulcerative proctitis or proctosigmoiditis. Dig Dis Sci.

