# Supplementary material for: Complete mucosal healing of distal lesions induced by twice-daily budesonide 2-mg foam promoted clinical remission of mild-to-moderate ulcerative colitis with distal active inflammation: double-blind, randomized study
Source: J Gastroenterol. 2017 Aug 4;53(4):494–506. doi: 10.1007/s00535-017-1376-4 (PMC5866841; doi:10.1007/s00535-017-1376-4)
Supplement: Supplementary file 1 — Supplementary material 1 (PDF 378 kb) [file 535_2017_1376_MOESM1_ESM.pdf]

Supplementary materials

(a)

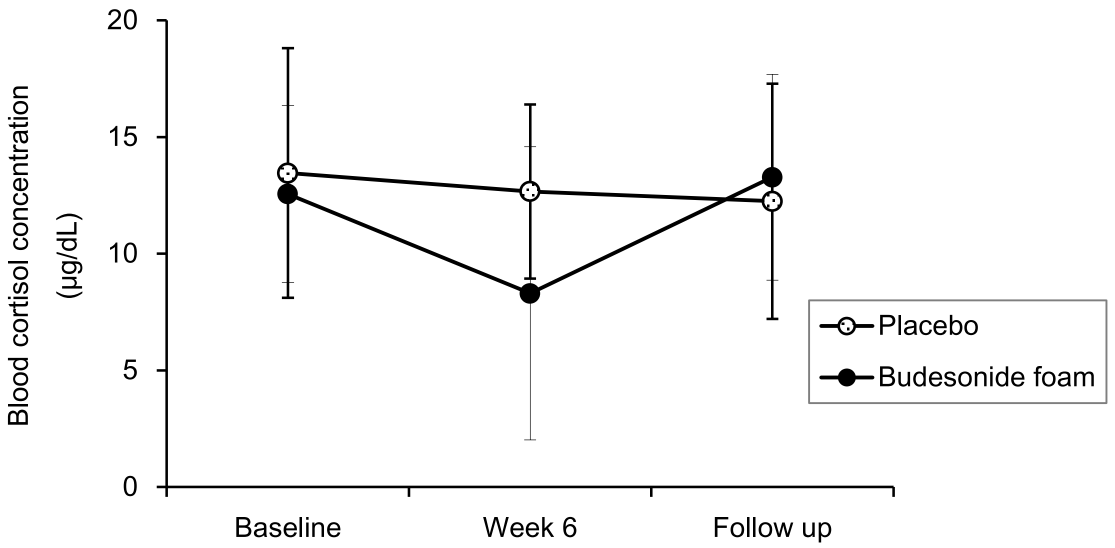

(b)

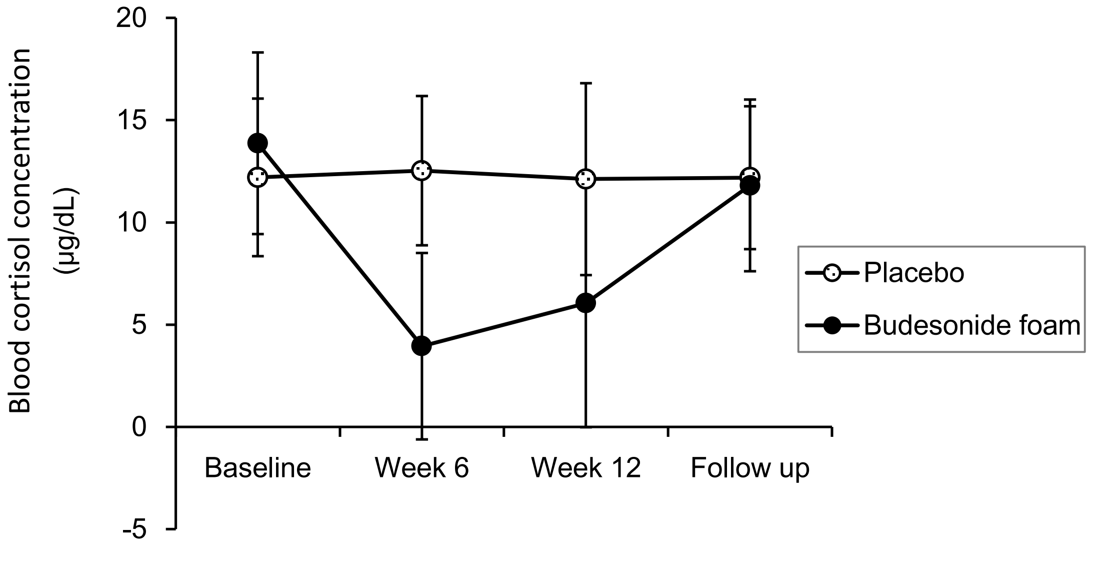

Figure S1. Mean morning plasma cortisol concentrations

(a) 6-week treatment, (b) 12-week treatment.

Data presented are mean and standard deviation.

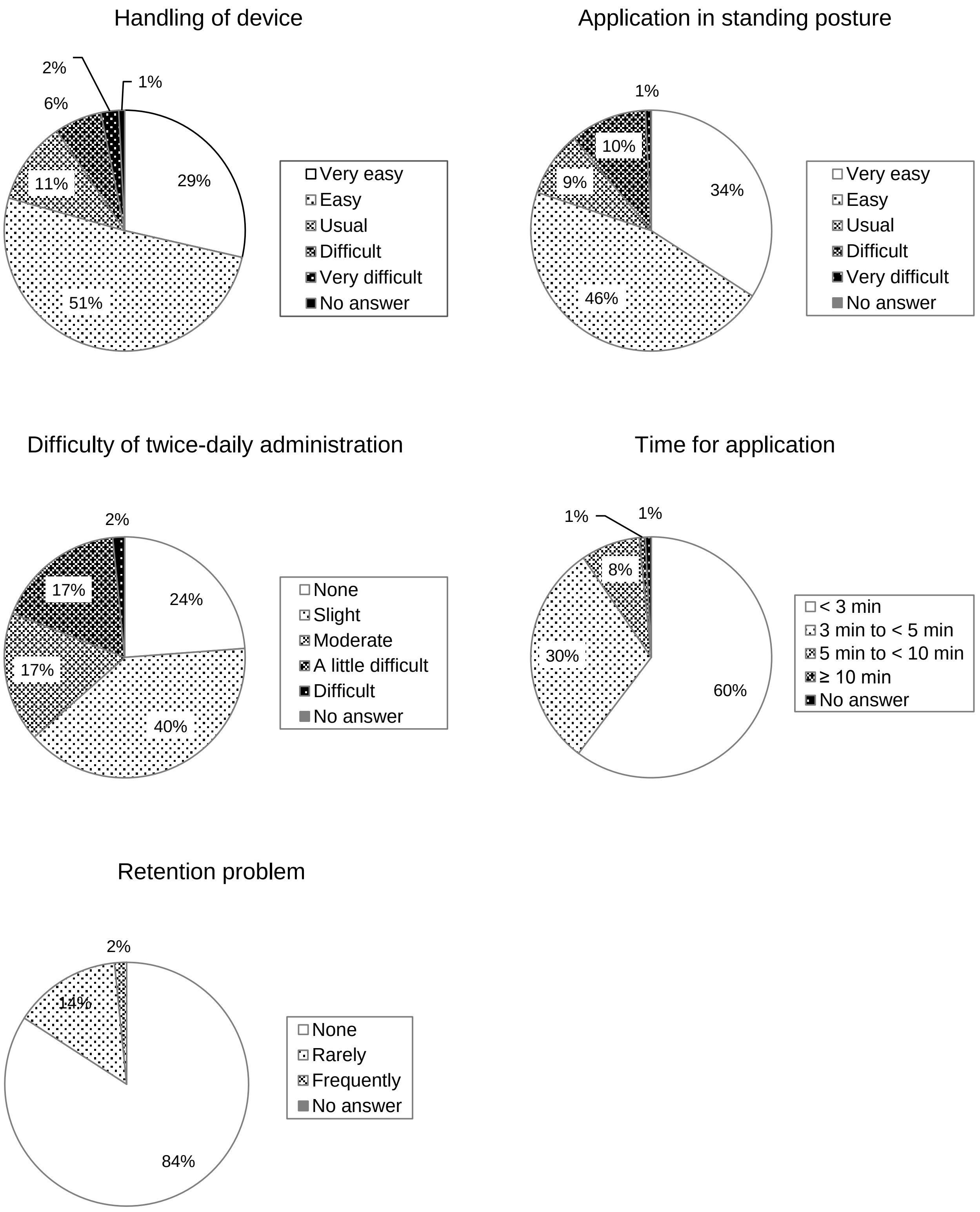

Figure S2. Patients' acceptance

All 126 patients were asked to complete questionnaires on general problems regarding the treatment at week 6 or at withdrawal visit.

Table S1. Other efficacy endpoints

	Placebo (N = 62)	Budesonide foam (N = 64)	Odds ratio	P value[a]
Endoscopic subscore ≤1, n (%)	22 (35.5)	48 (75.0)	5.716	< 0.0001
MMDAI[b] ≤1, n (%)	3 (4.8)	22 (34.4)	12.406	0.0002
Rectal bleeding subscore of 0, n (%)				
Week 2	15 (23.7)	34 (53.1)	3.801	0.0015
Week 4	19 (32.2)	40 (65.6)	4.510	0.0003
Week 6	24 (42.9)	41 (68.3)	3.090	0.0053

[a]Statistical analyses were performed at the level of significance of 0.05 (two-sided).

[b]MMDAI, Modified Mayo Disease Activity Index;
